# Multi-UAV Area Coverage Based on Relative Localization: Algorithms and Optimal UAV Placement

**DOI:** 10.3390/s21072400

**Published:** 2021-03-31

**Authors:** Ziyong Zhang, Xiaoling Xu, Jinqiang Cui, Wei Meng

**Affiliations:** 1 The Guangdong Province Key Laboratory of Intelligent Decision and Cooperative Control, School of Automation, Guangdong University of Technology, Guangzhou 510006, China; zhang_ziyong@163.com (Z.Z.); xiaolingandyou@163.com (X.X.); 2 Peng Cheng Laboratory, Shenzhen 518055, China; cuijq@pcl.ac.cn

**Keywords:** coverage control, ultra-wide band (UWB) sensors, relative localization, unmanned aerial vehicles (UAVs)

## Abstract

This paper is concerned with relative localization-based optimal area coverage placement using multiple unmanned aerial vehicles (UAVs). It is assumed that only one of the UAVs has its global position information before performing the area coverage task and that ranging measurements can be obtained among the UAVs by using ultra-wide band (UWB) sensors. In this case, multi-UAV relative localization and cooperative coverage control have to be run simultaneously, which is a quite challenging task. In this paper, we propose a single-landmark-based relative localization algorithm, combined with a distributed coverage control law. At the same time, the optimal multi-UAV placement problem was formulated as a quadratic programming problem by compromising between optimal relative localization and optimal coverage control and was solved by using Sequential Quadratic Programming (SQP) algorithms. Simulation results show that our proposed method can guarantee that a team of UAVs can efficiently localize themselves in a cooperative manner and, at the same time, complete the area coverage task.

## 1. Introduction

In recent years, unmanned aerial vehicle (UAV)-related research has attracted much attention. UAVs have many potential applications, such as area surveillance, and search and rescue [1,2,3,4,5]. Collaborative multi-UAV systems can significantly improve the work efficiency, especially when they perform challenging and complex tasks, such as target search in dangerous environments, i.e., a seismic region, or monitoring a large area-of-interest [6,7,8,9]. Area coverage is a typical fundamental application problem for multi-UAV systems, and the coverage problem is optimizing notions of quality-of-service (QoS) provided by an adaptive sensor network in a dynamic environment [10,11]. In real applications, dynamic environments, uncertain UAV locations, etc. will make the area coverage task more difficult [12,13,14,15,16]. The pioneer work in distributed coverage control was performed by J. Cortes [1]. In this work, the authors formulated the coverage control as a geographical optimization problem and derive the optimal decentralized. In [14], to make multi-UAVs cooperatively complete the coverage task more efficiently, the authors proposed a distributed coverage algorithm for a group of UAVs to cover a convex region with average Voronoi partition, which aims to ensure that the areas of all Voronoi partitions are identical. In [15], a distributed probability-based cooperative search method with Bayesian updating rule was addressed. Within that, a consensus-like fusion scheme was proposed for multiagent map fusion. The authors proved that all the individual probability maps converge to the same one that reflects the true existence or nonexistence of targets within each cell. In [17], Song et al. studied the coverage control problem for mobile sensor networks with limited communication ranges. The goal of the problem was to minimize a coverage cost function, which indicates the largest arrival time from the mobile sensor network to the points on a circle. In [18], the authors considered the heterogeneous coverage control problem, where the different density functions serve as a way to both abstract and encapsulate different sensing capabilities.

In the case that the UAVs’ location is not known a priori, the techniques of positioning already exist in both simulation and actual flight [19,20,21,22,23,24,25,26,27,28,29]. In [19], the authors proposed a method of passive detection of a moving aerial target by using heterogeneous satellites. In [20], methods of mode transition in the integrated navigation based on heterogeneous sensor integration were presented. In [21], the authors developed a method to reliably track many small rigid bodies with identical motion-capture marker arrangements. In [22], the authors presented a technique for multi-UAV localization using ranging measurements from Two-Way Time-Of-Flight UWB transceivers. However, the agents here must depend on some external infrastructure for positioning, relative localization techniques can be adopted to solve the location-based tasks, and the techniques do not need external infrastructure. In [23], the authors proposed a visual relative location (RL) algorithm, which is based on pattern detection using onboard cameras, to estimate the position of their agents. Their algorithm uses a combination of flood-fill techniques, which provide both high speed and precision for RL, but the performance of the algorithm is limited by the range to the target and lighting condition. In [24], the authors proposed an RL algorithm by using distance and odometry measurements to study the single landmark docking problem. The proposed method can achieve both autonomous approaching and landing tasks in GPS-denied environments. This experiment proves the feasibility of the RL algorithm for position estimation by using a single landmark. In [25], the relative position between neighboring UAVs is obtained by using UWB and visual odometry. Combined with the extended Kalman filter (EKF) strategy, it is successfully used to compute the global position of UAVs. In [26], the authors proposed a consensus-like relative localization scheme to estimate the relative position between the agents; the numerical simulations show that the relative position estimate exponentially converges to its true value. However, there is no discussion on the influence of measurement noises. In [27], the authors fused the obtained direct and indirect RL estimates by using a consensus-based fusion method to generate the relative positions. Their proposed RL estimation was applied to formation control.

The abovementioned research works study the area coverage problem and multi-UAV RL problem separately. However, in many real environments, such as in GPS-less environments, most UAVs’ locations cannot be known continuously or known with uncertainty. In this case, we have to consider the area coverage and multi-UAV RL problem together, which is the main focus of this work. To be more specific, the locations of a team of UAVs are unknown a priori when they perform the area coverage tasks. The main challenging issue will fall on (1) how to obtain the UAVs’ location when they perform the coverage task and (2) how to compromise the efforts of locational optimization placement between the relative localization and area coverage.

We assume that only one UAV can access its global position. Distance measurements are available through the distributed networks by using UWB technology. The relative localization problem is solved by combining the UWB measurements and UAV’s odometry measurements. The distributed coverage placement algorithm is proposed in [1], which is widely used in area coverage problems. For compromising the efforts of locational optimization placement between the relative localization accuracy and area coverage placement, the control inputs of the UAVs are obtained by solving the optimal UAVs coverage placement problem under the motion and communication constraints. The coverage placement problem comprises the location uncertainty optimization problem and locational optimization problem, and the solving process will adapt sequential quadratic programming (SQP) methods. The contribution of this paper are summarized as follows:

1. We propose an algorithm for multi-UAV to achieve the coverage task collaboratively based on the relative localization.
2. The optimal multi-UAV coverage placement is obtained by considering the compromise between the coverage placement and the sensor placement.

The remainder of this paper is organized as follows. Section 2 provides our considered relative localization-based area coverage problem formulation. Section 3 proposes the localization algorithm-based RL for coverage task performing. Section 4 proposes optimal multi-UAV placement for improving the coverage performance. The simulation and analysis are proposed in Section 5. Finally, the conclusions are drawn in Section 6.

## 2. Problem Formulation

**Figure 1:**
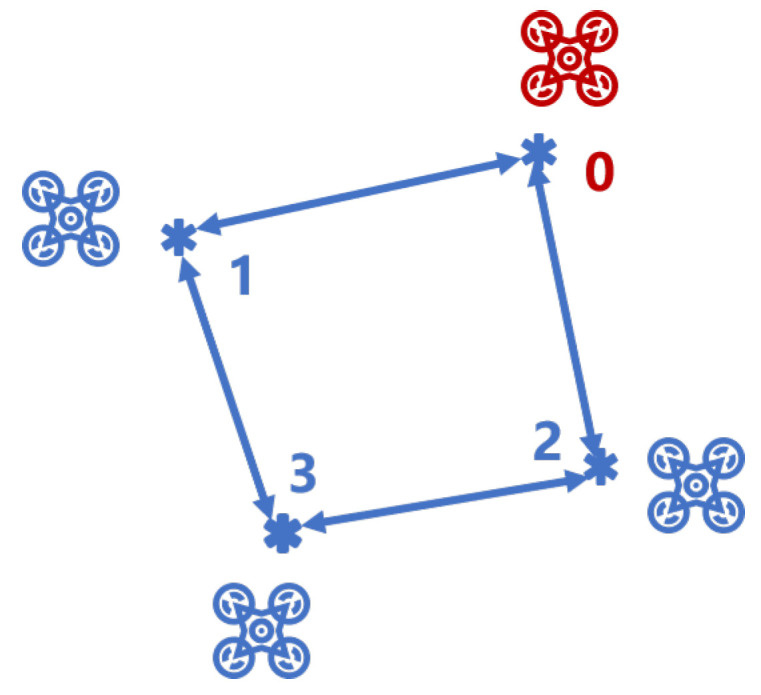
The communication topology of unmanned aerial vehicles (UAVs).

As mentioned in the Introduction section, we consider a relative localization-based area optimal multi-UAV coverage placement problem. Only one of the UAVs can access its global position, i.e., GPS signals, and other UAVs have to estimate their global position and perform the coverage task simultaneously. The position estimation algorithm uses the extended Kalman filter (EKF) strategy, which comprises cooperative localization and the motion of the UAVs. The cooperative localization is implemented in a decentralized way through a communication topology, as shown in Figure 1.

For the group of UAVs, the UAV that can access its global position is the leader, say node 0, and all others are followers labeled by nodes 1,...,n. UAVs exchange location estimates only with their neighbors through ultra-wide band (UWB) communication modules. The communication topology can be described by a undirected topology graph, i.e., the UAVs as the vertices of the graph. They only communicate with their neighbor UAVs that are within the communication range, as shown in Figure 1.

Every UAV is equipped UWB and visual odometry to measure the distance to their neighbors and their short-time movement displacement. Because of the large bandwidth (from 3.1 to 5.3 GHz), UWB can provide a reliable long-distance range with an accuracy of 10 cm. We use the DJI F450 (SZ DJI Technology Co., Ltd., Shenzhen, China) radio-controlled (RC) quadcopter, for which the dimensions are 50 cm × 50 cm × 20 cm. The test area is several tens of square meters, so an accuracy of 10 cm is complete enough. By using our RL algorithm, which will be presented in Section 3, the measurement can be used to compute the relative position between UAV and its neighbors. After obtaining their relative position, the UAVs can estimate their global position by exchanging local information with their neighbors. We can calculate the control inputs for every UAV to obtain the optimal multi-UAV area coverage placement. More details will be addressed in Section 4.

We use $p_{i,k}=\left(x_{i,k},y_{i,k}\right)$ to represent the global position of UAV *i* at time step *k*. $\chi_{k}^{i,j}=\left(x_{i,k}-x_{j,k},y_{i,k}-y_{j,k}\right)$ denote the estimation of relative position between UAVs *i* and *j*. $\left|\right|\chi_{k}^{i,j}\left|\right|=\sqrt{{\left(x_{i,k}-x_{j,k}\right)}^{2}+{\left(y_{i,k}-y_{j,k}\right)}^{2}}$ represents the relative distance between UAVs *i* and *j*.

The locational optimization function of coverage control is defined as

(1) $$H\left\{P_{k},W_{k}\right\}=\sum_{i=0}^{n}\int_{W_{i,k}}||q-p_{i,k}{\left|\right|}_{2}^{2}dq.$$

To find an optimal locational configuration of all UAVs that minimizes the locational cost function, with H representing the set of UAVs’ configuration, which includes the positions and the Voronoi partitions of multi-UAVs, let Q be a convex polytope in $R^{N}$ and set $P_{k}=\left(p_{0,k},p_{1,k},...,p_{n,k}\right)$ as the location of UAVs, which each move in Q. *q* is a point in Voronoi partition and the partition set is described by

(2) $$W_{k}=\left\{W_{1,k},W_{2,k},...,W_{i,k},...,W_{n,k}\right\},$$

where $W_{i,k}$ denotes the Voronoi region generated by UAV *i* at time *k* and two UAVs are defined as neighbors when their Voronoi regions are adjacent. The Voronoi region is shown as

(3) $$W_{i,k}=\{q\in Q|||q-p_{i,k}||<||q-p_{j,k}||,\forall i\neq j\},$$

The partial derivative of the cost function is shown as

(4) $$\begin{array}{cc} \frac{\partial H(P_{k},W_{k})}{\partial p_{i,k}} & =\int_{W_{i,k}}\frac{\partial ||q-p_{i,k}{\left|\right|}_{2}^{2}}{\partial p_{i,k}}dq\\ \; & =2\times M_{W_{i,k}}\left(p_{i,k}-C_{W_{i,k}}\right), \end{array}$$

where $M_{W_{i,k}}=\int_{W_{i,k}}dq$ is the mass of the Voronoi partition and $C_{W_{i,k}}=\frac{1}{M_{W_{i,k}}}\int_{W_{i,k}}qdq$ is the centroid of the Voronoi partition.

It has been proven in [1] that the configuration $\left\{p_{0},...,p_{n}\right\}=\left\{C_{W_{0}},...,C_{W_{n}}\right\}$ is optimal for H among all configurations in Q.

## 3. Materials and Methods

In this section, the localization of the multi-UAV-based RL algorithm and the process of coverage task performing are presented. The pseudo-code that integrates the whole process is shown at the end of the section.

During the process of a coverage task, the change in the UAV’s location is assumed to follow

(5) $${\hat{p}}_{i,k}={\hat{p}}_{i,k-1}+u_{i}\left(k-1\right),$$

where ${\hat{p}}_{i,k}$ denotes the position estimate of UAV *i* at time step *k* and $u_{i}\left(k-1\right)$ is the control input of UAVs. When all UAVs move to their targets, which are the corresponding centroids of their Voronoi partitions, UAVs exchange local information with their neighbors. The UAVs use the local information to update the Voronoi partition and target point in a given frequency. The group does not hover in place until the errors between the current estimated position and target position of every UAV are less than a predefined threshold.

In the process of the coverage task performance, the EKF strategy is considered to obtain optimal position estimation for every follower UAV. The strategy is composed of two parts, i.e., the first part is the EKF algorithm used for relative localization between each UAV and its neighbors, to compose the measurement equation of the second part. The second part is used for UAV’s global position estimation. The measurement equation of the UAV is obtained from the first part and motion equation of the UAVs.

For relative location EKF algorithm design, it is required to determine the relative state equation and relative observation equation. The relative state equation is given by

(6) $$\left\{\begin{array}{c} \chi_{k}^{i,j}=\chi_{k-1}^{i,j}+\Delta \chi_{k-1}^{i,j}+\xi_{k-1}^{i,j},\\ \Delta \chi_{k}^{i,j}=\Delta \chi_{k-1}^{i,j}+\upsilon_{k-1}^{i,j}+\delta_{k-1}^{i,j}, \end{array}\right.$$

where $\Delta \chi_{k-1}^{i,j}$ is displacement increment of UAV *i* and *j* from time step k−1 to *k* and $\upsilon_{k-1}^{i,j}$ is the difference of the displacement increment between UAV *i* and *j*. The terms $\xi_{k-1}^{i,j}$ and $\delta_{k-1}^{i,j}$ are the related Gaussian noise. On the other hand, the relative observation equation can be described as follows:

(7) $$\left\{\begin{array}{c} r_{k}^{i,j}=\left|\right|\chi_{k}^{i,j}\left|\right|+\eta_{k}^{i,j},\\ \phi_{k}^{i,j}=\Delta \chi_{k}^{i,j}+\nu_{k}^{i,j}, \end{array}\right.$$

where $r_{k}^{i,j}$ is the range measurement between UAV *i* and *j* at time step *k* and $\phi_{k}^{i,j}$ is the displacement measurement. The terms $\eta_{k}^{i,j}$ and $\nu_{k}^{i,j}$ are the corresponding Gaussian noise. Note that the only nonlinear part of the system comes from the relative range $r_{k}^{i,j}$, which needs to be linearized in the EKF:

(8) $$r_{k}^{i,j}={\hat{r}}_{k}^{i,j}+\frac{\chi_{k}^{i,j^{T}}}{\left|\right|\chi_{k}^{i,j}\left|\right|}\left(\chi_{k}^{i,j}-{\hat{\chi }}_{k}^{i,j}\right)+\eta_{k}^{i,j}.$$

The state can be rewritten as $s_{k}^{i,j}={\left[\begin{array}{cc} \chi_{k}^{i,j} & \Delta \chi_{k}^{i,j} \end{array}\right]}^{T}$. The state predict and update equation can be written as

(9) $${\hat{s}}_{k}^{i,j^{-}}=F_{{\hat{s}}_{k-1}^{i,j}}{\hat{s}}_{k-1}^{i,j^{+}}+\left[\begin{array}{c} 0\\ u_{i,k-1}-u_{j,k-1} \end{array}\right],$$

(10) $${\hat{s}}_{k}^{i,j^{+}}={\hat{s}}_{k}^{i,j^{-}}+K_{{\hat{s}}_{k}^{i,j}}\left(\left[\begin{array}{c} r_{k}^{i,j}\\ \phi_{k}^{i,j} \end{array}\right]-H_{{\hat{s}}_{k}^{i,j}}{\hat{s}}_{k}^{i,j^{-}}\right),$$

where

(11) $$F_{{\hat{s}}_{k-1}^{i,j}}=\left[\begin{array}{cccc} 1 & 0 & 1 & 0\\ 0 & 1 & 0 & 1\\ 0 & 0 & 1 & 0\\ 0 & 0 & 0 & 1 \end{array}\right],$$

(12) $$K_{{\hat{s}}_{k}^{i,j}}=\frac{\Sigma_{{\hat{s}}_{k}^{i,j}}^{-}H_{{\hat{s}}_{k}^{i,j}}^{T}}{H_{{\hat{s}}_{k}^{i,j}}\Sigma_{{\hat{s}}_{k}^{i,j}}^{-}H_{{\hat{s}}_{k}^{i,j}}^{T}+R_{{\hat{s}}_{k}^{i,j}}},$$

(13) $$\Sigma_{{\hat{s}}_{k}^{i,j}}^{-}=F_{{\hat{s}}_{k-1}^{i,j}}\Sigma_{{\hat{s}}_{k-1}^{i,j}}^{+}F_{{\hat{s}}_{k-1}^{i,j}}^{T}+Q_{{\hat{s}}_{k-1}^{i,j}},$$

(14) $$R_{{\hat{s}}_{k}^{i,j}}=E\left(\left[\begin{array}{c} \eta_{k}^{i,j}\\ \nu_{k}^{i,j} \end{array}\right]{\left[\begin{array}{c} \eta_{k}^{i,j}\\ \nu_{k}^{i,j} \end{array}\right]}^{T}\right),$$

(15) $$Q_{{\hat{s}}_{k-1}^{i,j}}=E\left(\left[\begin{array}{c} \xi_{k-1}^{i,j}\\ \delta_{k-1}^{i,j} \end{array}\right]{\left[\begin{array}{c} \xi_{k-1}^{i,j}\\ \delta_{k-1}^{i,j} \end{array}\right]}^{T}\right),$$

(16) $$H_{{\hat{s}}_{k}^{i,j}}=\left[\begin{array}{cc} {\frac{{\hat{\chi }}_{i,k}^{T}}{\left|\right|\chi_{i,k}\left|\right|}}_{1\times 2} & 0_{1\times 2}\\ 0_{2\times 2} & I_{2\times 2} \end{array}\right].$$

where ${\hat{s}}_{k}^{i,j}$ represents the estimation of $s_{k}^{i,j}$ and the symbols − and + represent the predicted state and updated state, respectively. $F_{{\hat{s}}_{k-1}^{i,j}}$ is the state transition matrix of the predicted equation and $K_{{\hat{s}}_{k}^{i,j}}$ is the Kalman gain. $\Sigma_{{\hat{s}}_{k}^{i,j}}$ is the covariance matrix of $s_{k}^{i,j}$. $R_{{\hat{s}}_{k}^{i,j}}$ and $Q_{{\hat{s}}_{k-1}^{i,j}}$ are the Gaussian noise matrices of EKF Equations (9) and (10). $H_{{\hat{s}}_{k}^{i,j}}$ is obtained by linearizing the relative range $r_{k}^{i,j}$ and the measurement equation of a UAV’s relative displacement. The updated value of state ${\hat{s}}_{k}^{i,j}$ can be obtained after computing Equations (9) and (10). The relative location covariance matrix update equation can be shown as

(17) $$\Sigma_{{\hat{s}}_{k}^{i,j}}^{+}=\left(I-K_{{\hat{s}}_{k}^{i,j}}H_{{\hat{s}}_{k}^{i,j}}\right)\Sigma_{{\hat{s}}_{k}^{i,j}}^{-}.$$

After obtaining the relative location of every UAV, the global position estimation can be derived by using the KF algorithm. The system model can be described as follows:

(18) $$\left\{\begin{array}{c} p_{i,k}=p_{i,k-1}+u_{i}\left(k-1\right)+\varphi_{i,k-1},\\ z_{i,k}=\sum_{j\in N_{i}}C_{k}^{i,j}\left({\hat{p}}_{j,k}+\chi_{k}^{i,j}\right)+\psi_{i,k}, \end{array}\right.$$

where $C_{k}^{i,j}=\frac{Tr({\left(\Sigma_{{\hat{p}}_{j,k}}+\Sigma_{\chi_{k}^{i,j}}\right)}^{-1})}{\sum_{m\in N_{i}}Tr\left({\left(\Sigma_{{\hat{p}}_{m,k}}+\Sigma_{\chi_{k}^{i,m}}\right)}^{-1}\right)}$ is as belief weight for location estimation. The weight is obtained by calculating the trace of the estimation uncertainty matrix. $z_{i,k}$ represents the measurement equation used for global position estimation. $\psi_{i,k}$ is the measurement noise of UAV *i* and $E\left(\psi_{i,k}\psi_{i,k}^{T}\right)=\sum_{j\in N_{i}}C_{k}^{i,j}\left(\Sigma_{{\hat{p}}_{j,k}}+\Sigma_{\chi_{k}^{i,j}}\right)$. The state predicted and updated equations can be written as

(19) $$\begin{array}{cc} {\hat{p}}_{i,k}^{-} & ={\hat{p}}_{i,k-1}^{+}+u_{i}\left(k-1\right), \end{array}$$

(20) $$\begin{array}{cc} {\hat{p}}_{i,k}^{+} & ={\hat{p}}_{i,k}^{-}+K_{{\hat{p}}_{i,k}}\left(z_{i,k}-H_{{\hat{p}}_{i,k}}{\hat{p}}_{i,k}^{-}\right), \end{array}$$

where

(21) $$H_{{\hat{p}}_{i,k}}=\left[C_{k}^{i,1},C_{k}^{i,2},...,C_{k}^{i,j},...\right],$$

(22) $$K_{{\hat{p}}_{i,k}}=\frac{\Sigma_{{\hat{p}}_{i,k}}^{-}H_{{\hat{p}}_{i,k}}^{T}}{H_{{\hat{p}}_{i,k}}\Sigma_{{\hat{p}}_{i,k}}^{-}H_{{\hat{p}}_{i,k}}^{T}+R_{{\hat{p}}_{i,k}}},$$

(23) $$\Sigma_{{\hat{p}}_{i,k}}^{-}=\Sigma_{{\hat{p}}_{i,k-1}}^{+}+Q_{{\hat{p}}_{i,k-1}},$$

(24) $$R_{{\hat{p}}_{i,k}}=E\left(\psi_{i,k}\psi_{i,k}^{T}\right)=\sum_{j\in N_{i}}C_{k}^{i,j}\left(\Sigma_{{\hat{p}}_{j,k}}+\Sigma_{\chi_{k}^{i,j}}\right),$$

(25) $$Q_{{\hat{p}}_{i,k-1}}=E\left(\varphi_{i,k-1}\varphi_{i,k-1}^{T}\right).$$

where $K_{{\hat{p}}_{i,k}}$ is the Kalman gain of Equation (18) and $\Sigma_{{\hat{p}}_{i,k}}$ is the global position estimation matrix of UAV *i*. $R_{{\hat{p}}_{i,k}}$ and $Q_{{\hat{p}}_{i,k-1}}$ are Gaussian noise matrices of KF Equations (9) and (10). The global location covariance matrix update equation can be derived as

(26) $$\Sigma_{{\hat{p}}_{i,k}}^{+}=\left(I-K_{{\hat{p}}_{i,k}}H_{{\hat{p}}_{i,k}}\right)\Sigma_{{\hat{p}}_{i,k}}^{-}.$$

The process of simultaneous coverage task and relative localization is summarized in Algorithm 1 shown below. We also tested the algorithm in numerical simulation by using MATLAB and robot operating systems (ROS). The process noise and measurement noise of the environment are obtained by comparing the optitrack system measurements with the measurements of sensors equipped in UAVs. The experimental values are the mean of many tests to close the randomness of a real environment. The physical characteristics such as the velocity of UAVs, etc., are obtained from real flight and measurements. More details about the test can be seen in Section 5.
**Algorithm 1 **Area coverage placement algorithm based on relative localization1:**Before Performing Coverage Task**2:    Before performing coverage task, the global position estimation of UAVs which can not obtain its global position is randomly given and their estimation covariance have a larger initial value.3:**Performing Coverage Task**4:    Set difference between final online expected coverage point position and UAV *i* as err and positive threshold of the difference as ε.5:**while** perform coverage task **do**6:    Every UAV *i* receives the global position of neighboring UAVs to compute its Voronoi region and move to the target position obtained by the distributed control law and the optimal solution of constrained nonlinear optimization problem7:    Carry out relative location EKF8:    Predict ${\hat{s}}_{k}^{i,j^{-}}\leftarrow \left(8\right)$9:    Compute $H_{{\hat{s}}_{k}^{i,j}}\;K_{{\hat{s}}_{k}^{i,j}}\;\Sigma_{{\hat{s}}_{k}^{i,j}}^{-}\leftarrow \left(11\right)\left(12\right)\left(15\right)$10:    Update ${\hat{s}}_{k}^{i,j^{+}}\;\Sigma_{{\hat{s}}_{k}^{i,j}}^{+}\leftarrow \left(9\right)\left(16\right)$11:    Carry out position location KF12:    Predict ${\hat{p}}_{i,k}^{-}\leftarrow \left(13\right)$13:    Compute $H_{{\hat{p}}_{i,k}}\;K_{{\hat{p}}_{i,k}}\;\Sigma_{{\hat{p}}_{i,k}}^{-}\leftarrow \left(20\right)-\left(22\right)$14:    Update ${\hat{p}}_{i,k}^{+},\Sigma_{{\hat{p}}_{i,k}}^{+}\leftarrow \left(19\right)\left(25\right)$15:    if |err|<ε then break16:**end while**

## 4. Optimal Multi-UAV Placement for Coverage

After every UAV calculates its global position, they perform the coverage task cooperatively. However, it is well known that the performance of the RL algorithm is highly related to the relative geometries of the group of UAVs, which will heavily influence the precision of location estimates. If the control input of the UAVs is determined only by factors of coverage task, that may cause relative localization performance to worsen. Hence, the compromise between the relative localization sensor placement and area coverage placement is quite important.

To achieve the goal, the control input $u_{i}\left(k\right)$ is considered to be comprised of two parts. One is obtained by minimizing the locational optimization function $F^{\prime }\left(u_{i}\left(k\right)\right)$, which is used for area coverage. This part is represented by $u_{i}^{\prime }\left(k\right)$, and the corresponding cost function is formulated as follows:

(27) $$\min_{u_{i}^{\prime }\left(k\right)}F^{\prime }\left(u_{i}\left(k\right)\right)=\sum_{i=0}^{n}\int_{W_{i,k}}||q-{\hat{p}}_{i,k}{\left|\right|}_{2}^{2}dq.$$

The other part is denoted by $u_{i,k}^{\prime \prime }$, which is obtained by minimizing the trace of uncertainty matrix of location estimates:

(28) $$F^{\prime \prime }\left(u_{i}\left(k\right)\right)=\mathrm{Tr}\left(\Sigma_{{\hat{p}}_{i,k}}^{+}\right).$$

When optimizing the above localization-related cost function, UAVs’ motion and communication constraints have to be considered. The resulted constrained nonlinear optimization function can be formulated as follows:   

(29) $$\begin{array}{cc} \min_{u_{i}^{\prime \prime }\left(k\right)} & F^{\prime \prime }\left(u_{i}\left(k\right)\right)=\mathrm{Tr}\left(\Sigma_{{\hat{p}}_{i,k}}^{+}\right),\\ \;\mathrm{subject}\;\mathrm{to}\; & \end{array}$$

(30) $$\begin{array}{cc} \; & \left|\right|u_{i}\left(k\right)-u_{i}\left(k-1\right)\left|\right|\;\leq \;u_{max}, \end{array}$$

(31) $$\begin{array}{cc} \exists j\in N_{i}\; & \left|\right|{\hat{p}}_{i,k+1}-{\hat{p}}_{j,k+1}\left|\right|\;\leq \;m_{d}, \end{array}$$

(32) $$\begin{array}{cc} \forall j\in N_{i}\; & \left|\right|{\hat{p}}_{i,k+1}-{\hat{p}}_{j,k+1}\left|\right|\;\geq \;n_{d}, \end{array}$$

(33) $$\begin{array}{cc} x_{min} & \leq x_{{\hat{p}}_{i,k+1}}\leq x_{max}, \end{array}$$

(34) $$\begin{array}{cc} y_{min} & \leq y_{{\hat{p}}_{i,k+1}}\leq y_{max}, \end{array}$$

where the condition (30) presents the UAV input constraint, e.g., its turning rate constraint. The condition (31) addresses the case that UAV *i* has to maintain communication with at least one of its original neighbors. The condition (32) presents UAV *i* as maintaining collision avoidance distance $n_{d}$ with its neighbors. The conditions (33) and (34) present that UAV *i* will always move within the given region.

From the problem formulation (29)–(34), we can see that the optimization problem is nonlinear with linear and nonlinear constraints. Gradient methods cannot be directly used for this problem. Instead, the SQP algorithm can be utilized to solve such an optimization problem.

**Figure 2:**
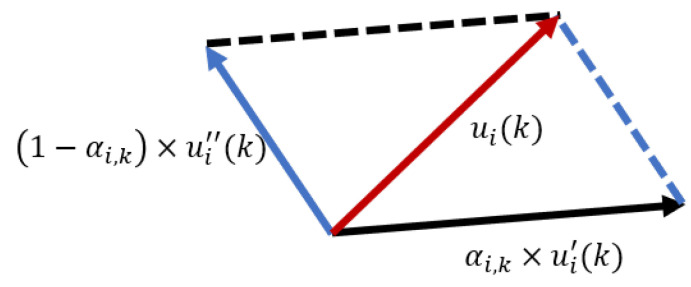
The composition of the control input.

The expected control input can be considered the join force using the coverage control and relative localization parts shown in Figure 2. The control input $u_{i}\left(k\right)$ of each part can be calculated by adjusting the weight $\alpha_{i,k}\left(0\leq \alpha_{i,k}\leq 1\right)$, which is given by

(35) $$u_{i}\left(k\right)=\alpha_{i,k}\times u_{i}^{\prime }\left(k\right)+\left(1-\alpha_{i,k}\right)\times u_{i}^{\prime \prime }\left(k\right),$$

where weight $\alpha_{i,k}$ is given by

(36) $$\begin{array}{cc} \alpha_{i,k} & =\frac{\mathrm{Tr}(\Sigma_{{\hat{p}}_{i,k}}^{+})}{\mathrm{Tr}(\Sigma_{{\hat{p}}_{i,k}}^{+})+T}-\frac{1}{2}, \end{array}$$

where *T* is a judgement threshold, which can be set according to the converge performance of the RL, i.e., when $\mathrm{Tr}(\Sigma_{p_{i,k}}^{+})$ drops to *T*.

Combined with Algorithm 1, the optimal RL-based coverage placement algorithm is summarized in Algorithm 2.
**Algorithm 2 **Optimal relative location (RL)-based coverage placement algorithm.1:**Performing Coverage Task**2:   Set difference between the position of final online expected coverage point and UAV *i* as err and positive threshold of the difference as ε3:**while** perform coverage task **do**4:    Communicate with all neighbours.5:    Compute $u_{i}^{\prime }\left(k\right),u_{i}^{\prime \prime }\left(k\right),\alpha \to u_{i}\left(k\right)$ (29)–(37)6:    Carry out relative location EKF7:    Update ${\hat{s}}_{k}^{i,j^{+}},\Sigma_{{\hat{s}}_{k}^{i,j}}^{+}$ (9)(16)8:    Carry out position location KF9:    Update ${\hat{p}}_{i,k}^{+},\Sigma_{{\hat{p}}_{i,k}}^{+}$ (19)(25)10:    if |err|<ε then break.11:**end while**

## 5. Simulation Results

In this section, the performance of the coverage algorithm proposed in this paper is evaluated using the simulation tests. The parameters used in the simulations are listed as follows: 4 UAVs are assigned randomly in the given square region for coverage task. The size of the coverage area is 15 m × 15 m. The UAVs move at an average speed of 0.2 m/s, and the communication frequency is 10 Hz. In the global position estimation KF algorithm, the process noise is set as $\varphi_{i,k-1}∼N\left(0,0.1^{2}\right)$. In the relative location estimation EKF algorithm, $\xi_{k-1}^{i,j}=\varphi_{i,k-1}-\varphi_{j,k-1}$ and $\delta_{k-1}^{i,j}∼N\left(0,0.02^{2}\right)$, and the displacement measurement noise $\eta_{k}^{i,j}∼N\left(0,0.01^{2}\right)$ and $\nu_{k}^{i,j}∼N\left(0,0.02^{2}\right)$. The velocity of UAVs and the communication frequency are obtained from our real experimental equipment. The gaussian noise of position estimation is obtained from our truly flight test. The approach we proposed has a complexity of $O(N^{3})$ because the matrix inverse calculation is involved in the process of position estimation. The average computing time ranged from 0.013 to 0.028 s, and the processer used was Intel i7-1165G7.

**Figure 3:**
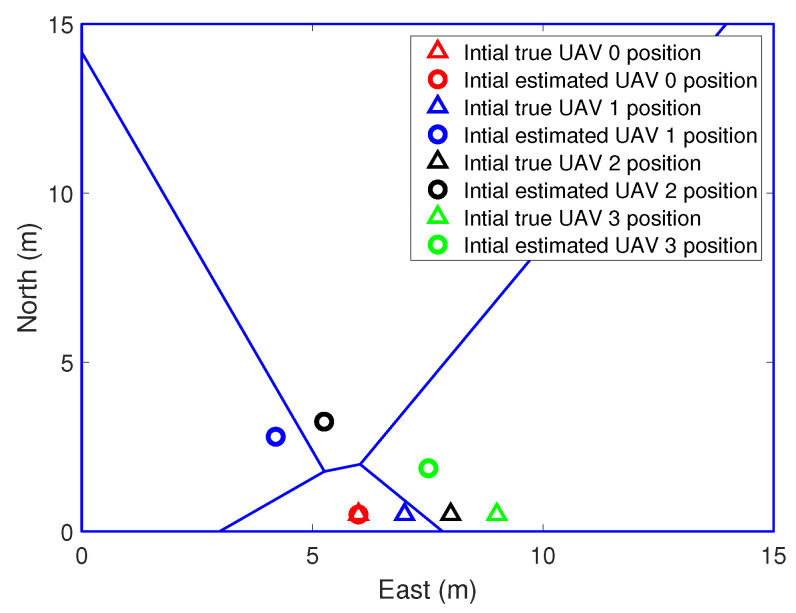
The process of coverage task performing, k = 0 s.

**Figure 4:**
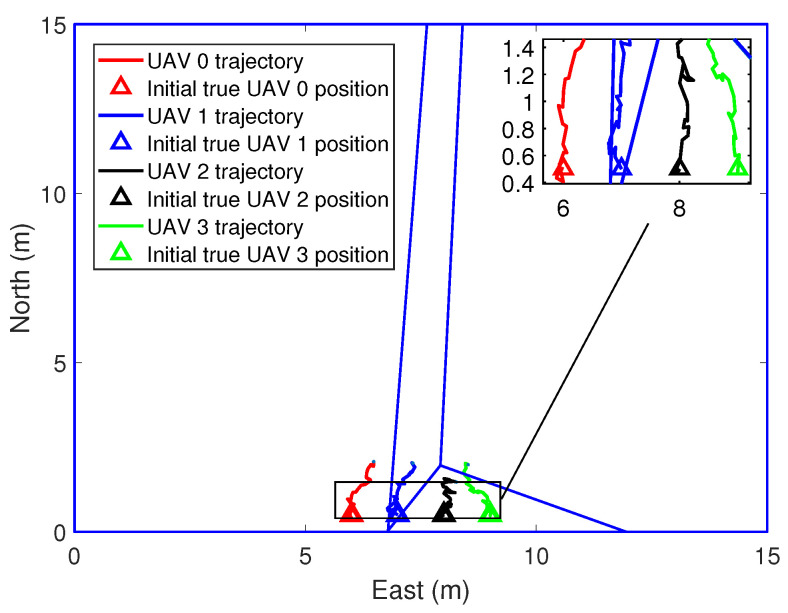
The process of coverage task performing, k = 20 s.

**Figure 5:**
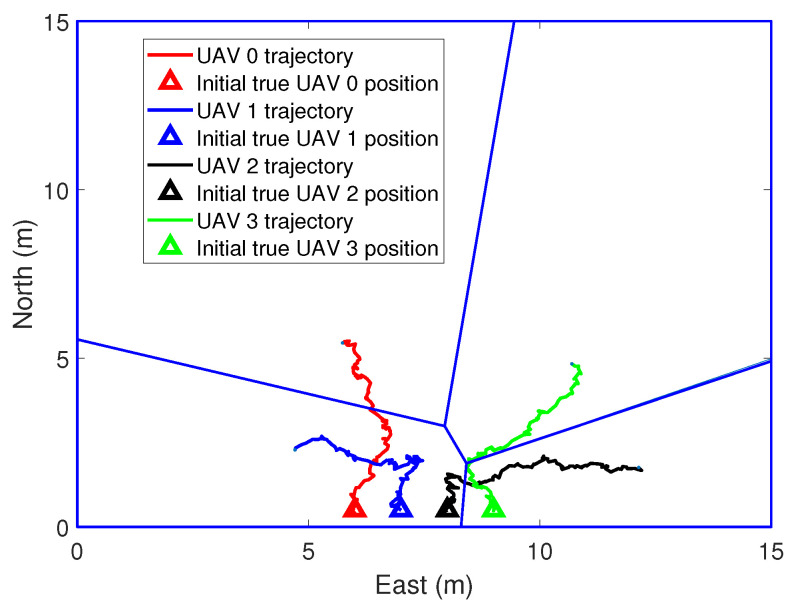
The process of coverage task performing, k = 100 s.

In the simulations, the real position and the initial position guess of the UAVs are shown in Figure 3. Voronoi partition is based on the estimated position of the UAVs. Figure 4 and Figure 5 show the initial to medium stages of the RL-based coverage placement. In the figures, a straight line is used to represent the boundary of the Voronoi partitions. Curves represent the flight trajectories of the UAVs. As we can see in Figure 4, the location estimation uncertainty of the UAVs is pretty large at the initial stage; hence, $u_{i,k}^{\prime \prime }$ dominates the control input and UAVs have to keep close to each other to ensure the precision of relative localization. With the execution of the coverage task, the covariance measure of the position estimation decreases gradually and then $u_{i,k}^{\prime }$ dominates the control input, as shown in Figure 5.

**Figure 6:**
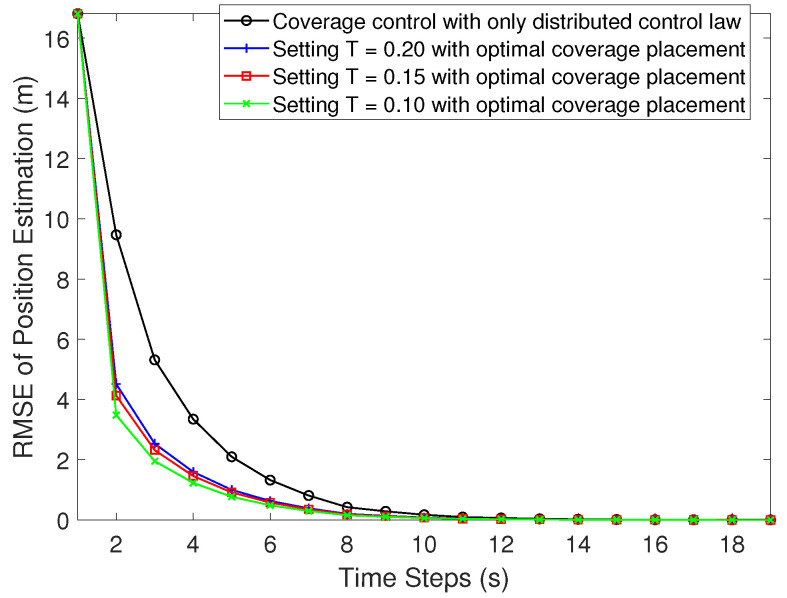
The root-mean-square error (RMSE) of a UAV’s position estimation.

The relative localization performance results are shown in Figure 6. The root-mean-square error (RMSE) is utilized as the localization accuracy metric. From the figure, we can see that the RMSE of the position estimate of follower UAVs converge to 0 gradually and remain accurate until convergence of the coverage task. We choose different values of *T* (threshold used in the weight setting in (36)). Compared with the one without considering the control input originated from the localization optimization, by using the join force strategy (35), localization performance is better.

**Figure 7:**
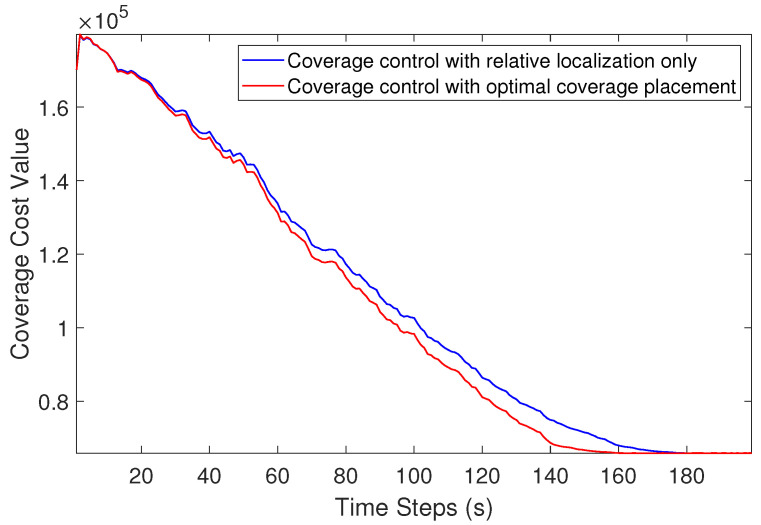
The change in coverage cost value.

We also compared the change in coverage cost value with other published approaches. Our work considers the coverage with relative localization of UAVs and optimal UAV placement. As shown in Figure 7, the coverage performance is better using the approach we proposed.

**Figure 8:**
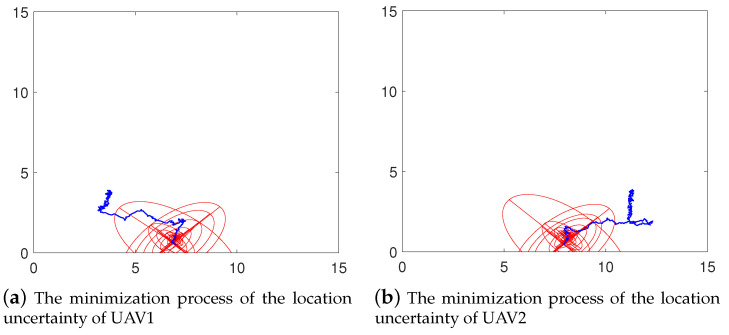

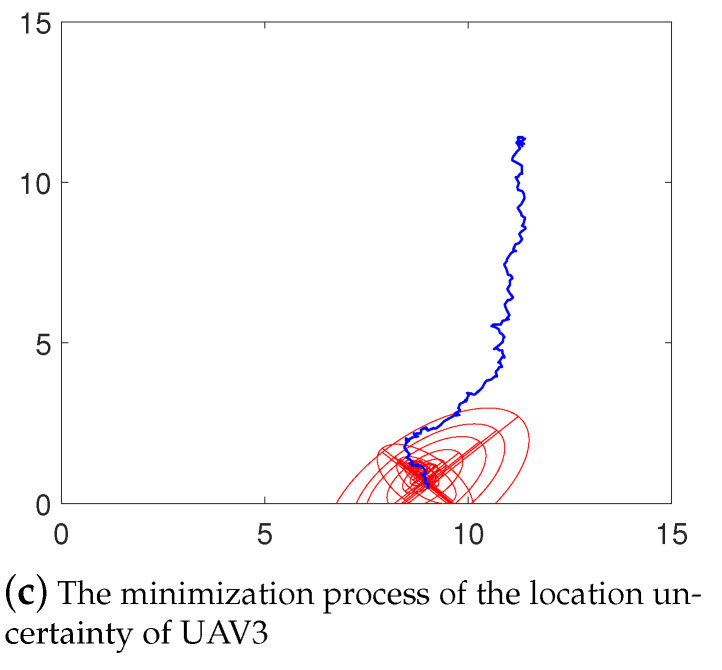
The minimization process of the location uncertainty. Blue lines represent the flight trajectory of the UAVs. The red ellipses represent the corresponding depict of the location uncertainty in each step.

The location uncertainty ellipse of the follower UAVs is shown in Figure 8. As we can see from the figures, the location uncertainties of follower UAVs is pretty large at the initial stage. Localization errors decrease gradually while performing cooperative localization.

**Figure 9:**
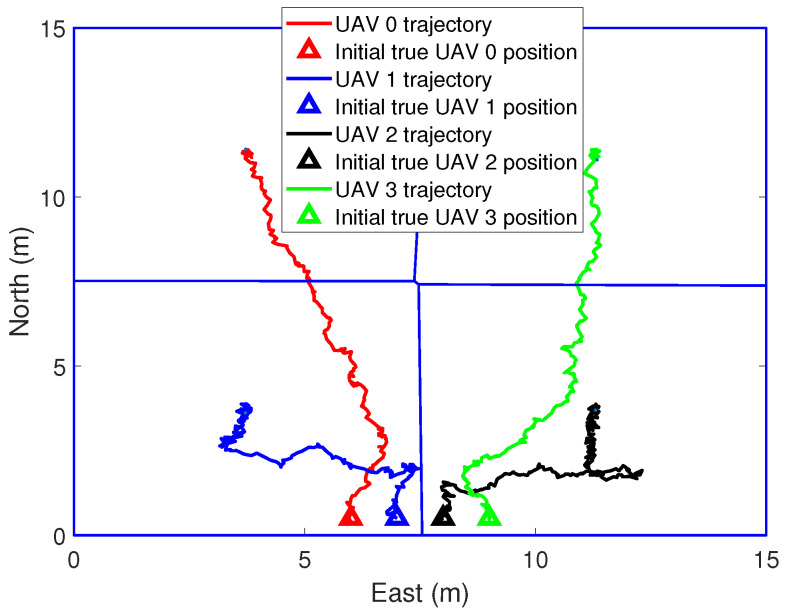
The process of coverage task performing, k = 200 s.

The overall process of the RL-based multi-UAV coverage task is shown in Figure 9, In the figure, the whole procedure is shown, i.e., from initial RL dominated stage to the final coverage control dominated stage. By using our proposed RL-based coverage control strategy, even with most of the UAVs having no location information or having large uncertainties a priori, UAVs can estimate their location using RL methods and can simultaneously fight the centroid of their Voronoi regions in a given area, as shown in [1], and complete the area optimal placement coverage task.

## 6. Conclusions

In this paper, we proposed a coverage algorithm based on relative localization to perform the coverage task for the group of UAVs in the case that only one UAV knows its position in advance. Because the performance of coverage placement and the position estimation of the UAVs influence each other, the proposed optimal multi-UAV placement strategy takes into account both RL localization geometry optimization and coverage placement optimization. The simulation results show that the coverage placement algorithm based on relative localization is able to achieve the coverage task and that the coverage performance is improved by the proposed optimal multi-UAV placement strategy. Our future work will include a discussion for limitations in terms of energy and computing resources and the algorithm test in a real experiment.

## Data Availability

Not applicable.
